# Supplementary material for: Solution Combustion Synthesis of Hafnium-Doped Indium Oxide Thin Films for Transparent Conductors
Source: Nanomaterials (Basel). 2022 Jun 23;12(13):2167. doi: 10.3390/nano12132167 (PMC9268072; doi:10.3390/nano12132167)
Supplement: Supplementary file 1 [file nanomaterials-12-02167-s001.zip › nanomaterials-1761394-supplementary.pdf]

nanomaterials

Supplementary Material

Solution Combustion Synthesis of Hafnium-Doped Indium Oxide Thin Films for Transparent Conductors

Rita Firmino, Emanuel Carlos *, Joana Vaz Pinto, Jonas Deuermeier, Rodrigo Martins, Elvira Fortunato, Pedro Barquinha * and Rita Branquinho *

CENIMAT|i3N, Department of Materials Science and CEMOP/UNINOVA, NOVA School of Science and Technology, NOVA University of Lisbon, 2829-516 Caparica, Portugal; r.firmino@campus.fct.unl.pt (R.F.); jdvp@fct.unl.pt (J.V.P.); j.deuermeier@fct.unl.pt (J.D.); rfpm@fct.unl.pt (R.M.); emf@fct.unl.pt (E.F.)

* Correspondence: e.carlos@fct.unl.pt (E.C.); pmcb@fct.unl.pt (P.B.); ritasba@fct.unl.pt (R.B.)

The supplementary material contains relevant data related to the characterization of dopant quantity and post-deposition treatments of In_2O_3 thin films developed in this work. Figure S1 and Table S1 display the FTIR spectra of solutions and doped thin films. Figure S2 shows SEM images of thin films with different annealing conditions and doping. XPS analysis is presented on Figure S3 and S4, and Table S2 and S3. For the different annealing condition study thickness (Table S4), transmittance (Figure S5), XRD diffractogram (Figure S6), AFM images (Figure S7) and atomic ratio (Figure S8) are also presented. Table S5 shows the results electrical properties of Hf-doped thin films prepared by various methods.

FTIR Analysis of Precursor Solutions and Hf-Doped In_2O_3 Thin Films

Figure S1 depicts the FT-IR spectra of undoped and 0.5 M % Hf-doped indium oxide solutions and thin films for each molar ratio studied. Most of the vibration bands in precursor solutions

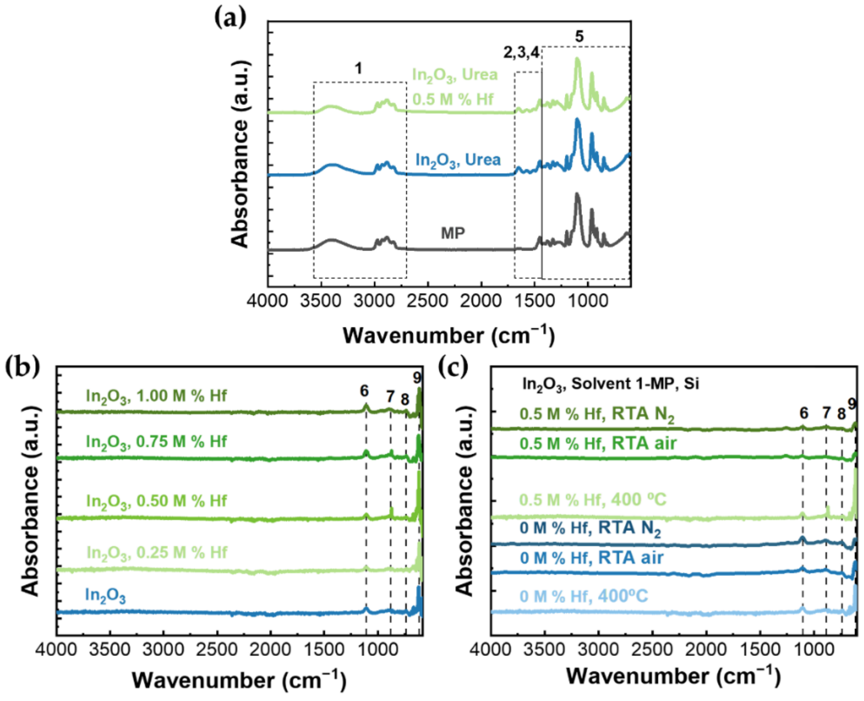

Figure S1. FTIR spectra of (**a**) In_2O_3 solutions with urea, and combustion doped 0.5 M % of Hf; (**b**) indium oxide thin films doped with different hafnium quantities, annealed at 400 °C in air; (**c**) undoped and 0.5 M % Hf-doped indium oxide thin films, annealed at 400 °C in air.

Figure. S1a are expected for 1-methoxy-2-propanol, that's the case of the ranges numbered as 1 (3500-2800 cm^{-1}) and 5 (1450-850 cm^{-1}). Although the peak at 1110 cm^{-1} could be also attributed to Si-O bond, from the substrate. Peaks labeled as 2 (1650 cm^{-1}) and 3 (1575 cm^{-1}) are attributed to C-O and N-H vibrations from urea.[1] The peak 4 present at 1510 cm^{-1} can be correlated with N-O vibrations, originated from the indium precursor.

Table S1. Characteristic absorbance peaks and associated vibrational modes of the corresponding chemical bonds for the spectrum of indium oxide thin films on silicon.

Number	Position (cm^{-1})	Mode Type	Chemical Bond
**6**	1110	Transverse Optical Stretching	Si-O
**7**	896	Bending	In-O
**8**	736	Stretching	In-O
**9**	613	Bending	In-O

As reflected in Figure S1b and S1c, after correction of the atmosphere contribution, all samples seem to have similar behaviors, meaning that the different dopant quantities did not prevent the reaction to fully occur. It is possible to confirm that the reaction occurred to its full extend as none of the organic absorbance peaks presented in the solution spectra are visible, Figure S1b and S1c. The absorbance peaks that appear between 890 and 610 cm^{-1} are characteristic of vibrational modes of indium, as shown in Table S1.[2]

SEM Images

Surface morphology analysis was performed by SEM, and the results are shown in Figure S2. All annealing conditions presented a granular, uniform, and crack-free surface.

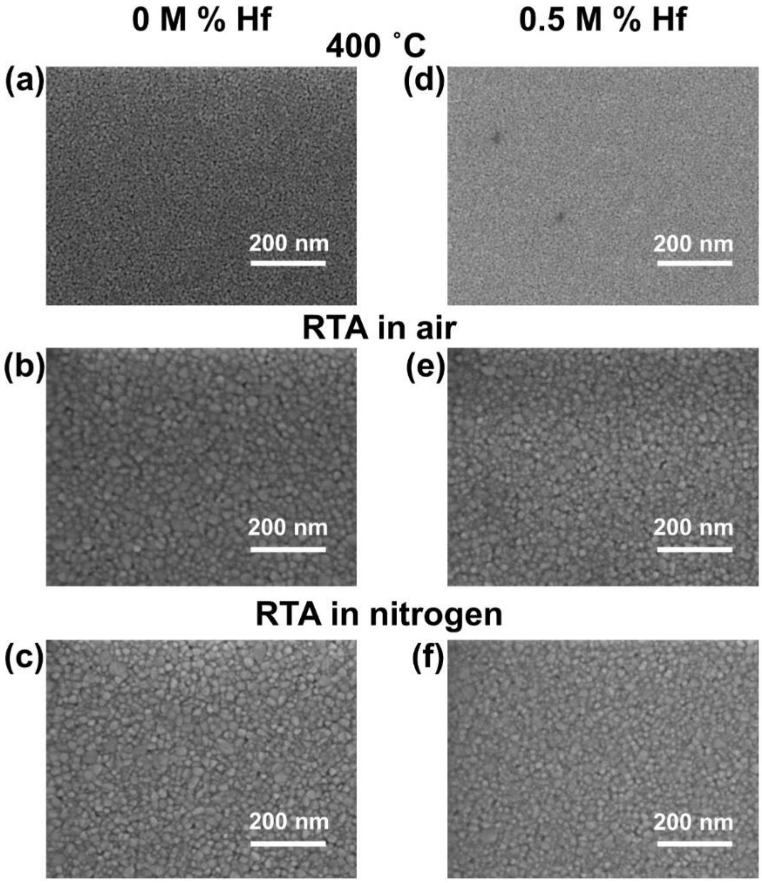

Figure S2. SEM surface images of (**a**–**c**) undoped and (**d**–**f**) Hf-doped In_2O_3 thin films annealed at (**a**,**d**) 400 °C (ambient conditions), (**b**,**e**) RTA in air and (**c**,**f**) RTA in nitrogen at 600 °C.

XPS Analysis

XPS spectra of undoped and Hf-doped In_2O_3, at different dopant concentrations and annealing conditions are presented on Figure S3 and S4.

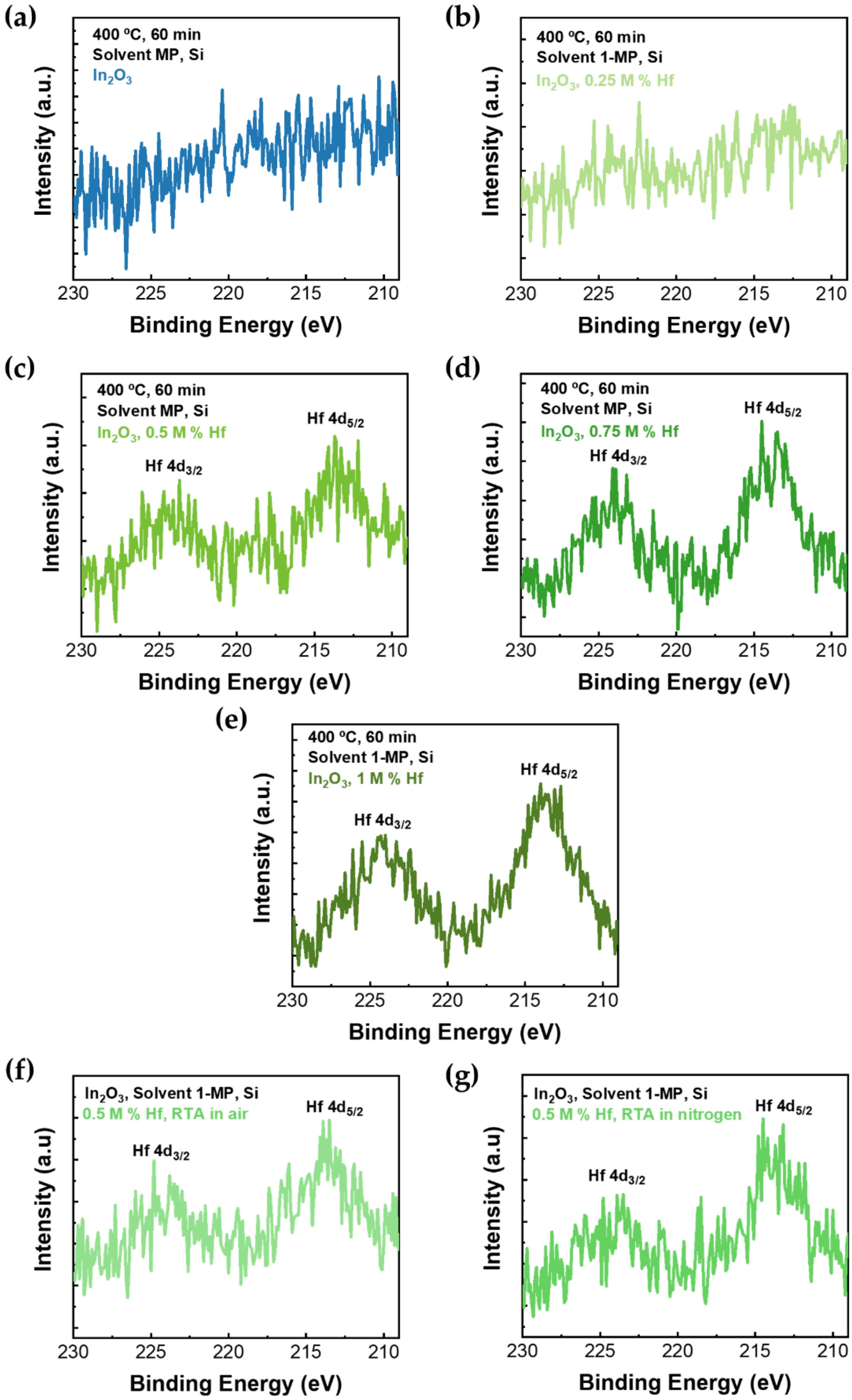

Figure S3. Hf 4*d* core-level spectra of In_2O_3 thin films (**a**) undoped and doped with (**b**) 0.25 M % Hf, (**c**) 0.5 M % Hf, (**d**) 0.75 M % Hf and (**e**) 1 M % Hf annealed at 400 °C in air. Hf 4*d* core-level spectra of 0. 5 M % Hf doped In_2O_3 thin films annealed in RTA in (**f**) air and (**g**) nitrogen at 600 °C for 10 min.

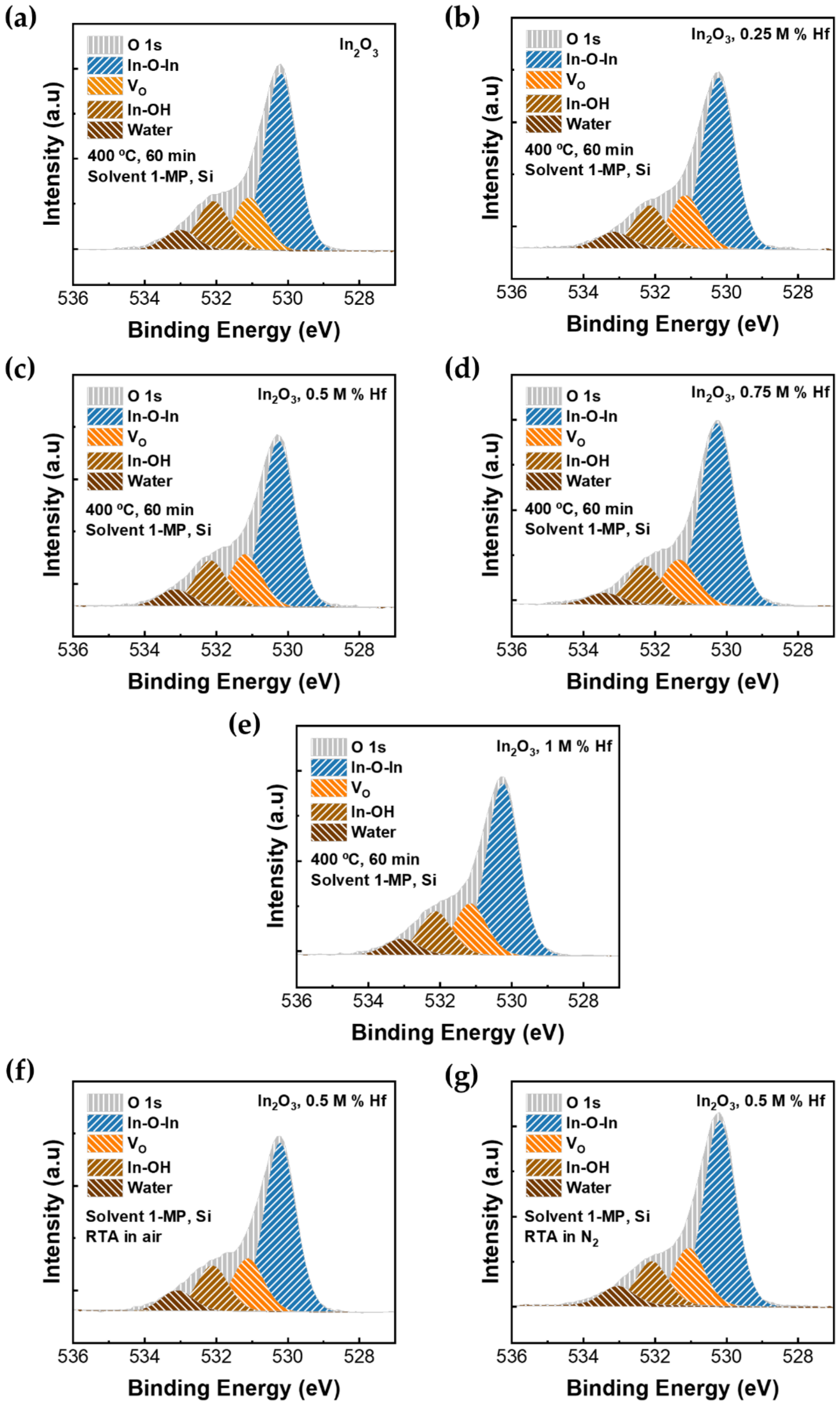

Figure S4. O 1*s* core-level spectra of In_2O_3 thin films (**a**) undoped and doped with (**b**) 0.25 M % Hf, (**c**) 0.5 M % Hf, (**d**) 0.75 M % Hf and (**e**) 1 M % Hf annealed at 400 °C in air. O 1*s* core-level spectra of 0. 5 M % Hf doped In_2O_3 thin films annealed in RTA in (**f**) air and (**g**) nitrogen at 600 °C for 10 min.

Table S2. Peak area, in %, of each peak present in core-level O 1s peak for each M % of Hf doping in In_2O_3 thin films.

M % Hf	Area (%)			
	**In-O-In**	**V_o**	**In-OH**	**H_2O**
0	59.44	17.66	16.29	6.61
0.25	60.56	18.76	15.05	5.63
0.5	59.02	18.65	16.29	6.04
0.75	65.26	16.25	14.28	4.72
1	60.66	18.23	15.39	5.72

Table S3. Area, in %, of each of the peaks present in core-level O 1s peak for 0.5 M % Hf doped-In_2O_3 thin films annealed in different conditions.

Annealing	Area (%)			
	**In-O-In**	**V_o**	**In-OH**	**H_2O**
400 °C	59.02	18.65	16.29	6.04
RTA in air	58.48	18.38	15.88	7.26
RTA in N_2	60.61	18.82	14.35	6.22

Characterization of Films with Different Annealing Conditions

Table S4. Thickness of undoped and Hf-doped In_2O_3 thin films with different annealing conditions.

Dopant	Annealing		
	**400 °C on Hotplate**	**RTA in Air**	**RTA in N_2**
-	104.0 ± 0.6 nm	79.12 ± 0.4 nm	79.34 ± 0.4 nm
0.5 M % Hf	110.9 ± 1.3 nm	92.22 ± 0.5 nm	92.09 ± 0.3 nm

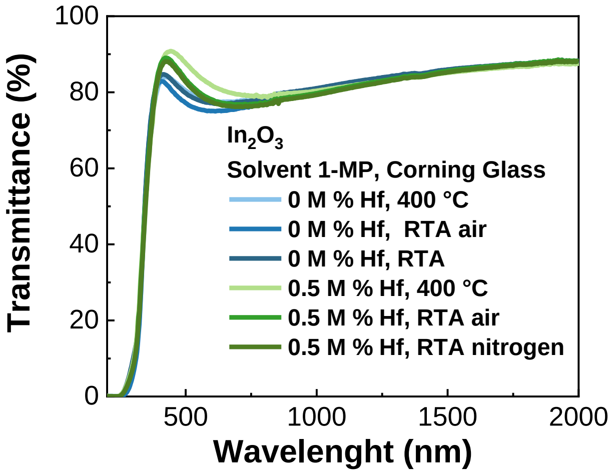

Figure S5. Transmittance spectra of undoped and 0.5 M % Hf- doped In_2O_3 thin films.

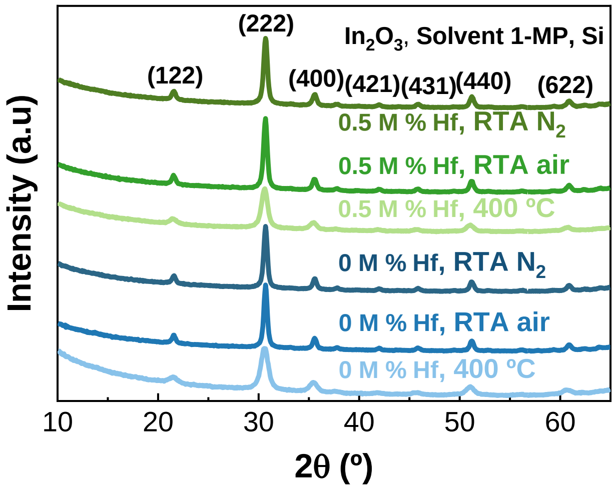

Figure S6. XRD diffractograms of undoped and Hf-doped In_2O_3 thin films, with different post-deposition treatments.

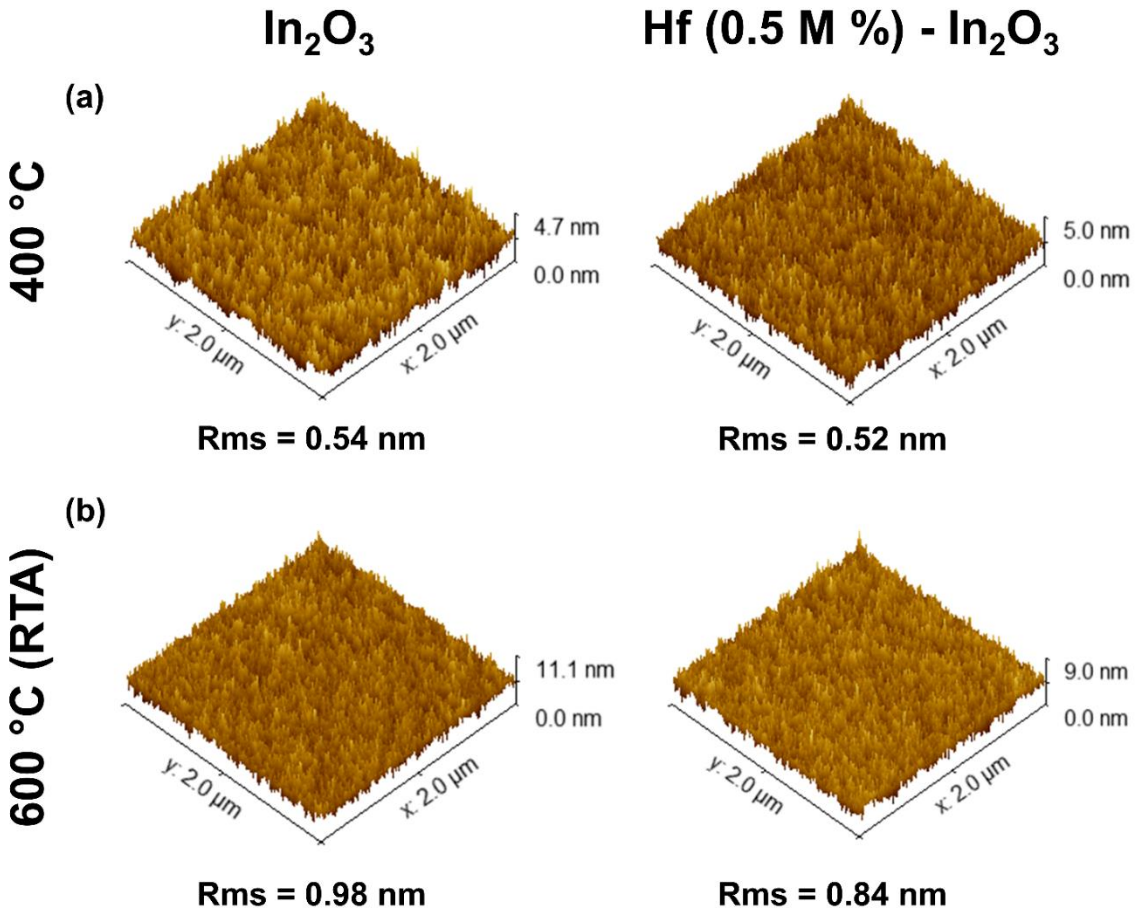

Figure S7. AFM images (2 × 2 μm²) of the undoped and doped indium oxide thin films in air (**a**) in a hotplate at 400 °C and (**b**) in RTA at 600 °C.

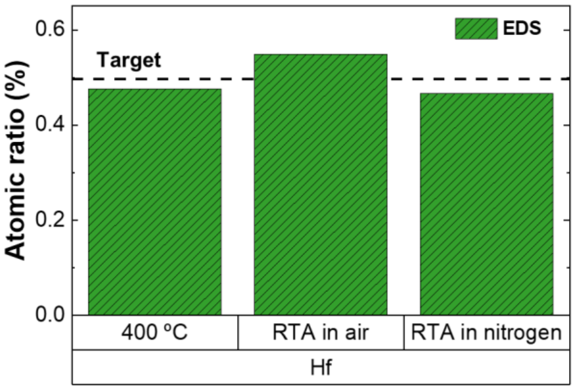

Figure S8. Atomic ratio (%) of 0.5 M % Hf-doped In_2O_3 thin films obtained throughout EDS.

Hafnium Doped TCOs

Table S5. Electrical properties of Hf-doped transparent conductive thin films prepared by various methods.

Year	TCO	Process	$T_{550\ nm}$ (%)	ϱ (Ω.cm)	N ($\times 10^{20} cm^{-3}$)	μ ($cm^2/V\ s$)
2012 [3]	Hf (3.3 at.%) - ZnO	ALD [1]	80	6.0×10^{-4}	3.7	20.0
2013 [4]	Hf (4.6 at.%) - ZnO	ALD [1]	-	1.6×10^{-3}	3.5	12.5
2015 [5]	Hf (3 at %) - ZnO	Dip-coating	75	5.6×10^{-3}	0.5	2.5
2017 [6]	Hf (2.8 wt%) – In_2O_3	RF Sputtering	82	3.8×10^{-4}	4.1	50.7
2019 [7]	1:1 Hf:ZnO cycle ratio	ALD [1]	79	1.6×10^{-3}	2.4	16.0
2020 [8]	Hf (1 wt%) – In_2O_3	RF Sputtering	80	3.6×10^{-5}	45	39.5
**This work**	Hf (0.5 M %) - In_2O_3	Spin-coating	80	4.0×10^{-3}	0.8	21.0

[1] Atomic Layer Deposition.

References

1. Manivannan, M.; Rajendran, S. Investigation of inhibitive action of urea-Zn2+ system in the corrosion control of carbon steel in sea water. *Int. J. Eng. Sci. Technol.* **2011**, *3*, 8038–8060.
2. Ayeshamariam, A.; Bououdina, M.; Sanjeeviraja, C. Optical, electrical and sensing properties of In2O3 nanoparticles. *Mater. Sci. Semicond. Process.* **2013**, *16*, 686–695, doi:10.1016/J.MSSP.2012.12.009.
3. Ahn, C.H.; Kim, J.H.; Cho, H.K. Tunable Electrical and Optical Properties in Composition Controlled Hf:ZnO Thin Films Grown by Atomic Layer Deposition. *J. Electrochem. Soc.* **2012**, *159*, H384–H387, doi:10.1149/2.026204JES/XML.
4. Geng, Y.; Xie, Z.Y.; Yang, W.; Xu, S.S.; Sun, Q.Q.; Ding, S.J.; Lu, H.L.; Zhang, D.W. Structural, optical, and electrical properties of Hf-doped ZnO films deposited by atomic layer deposition. *Surf. Coatings Technol.* **2013**, *232*, 41–45, doi:10.1016/J.SURFCOAT.2013.04.050.
5. Wang, F.; Zhao, X.; Duan, L.; Wang, Y.; Niu, H.; Ali, A. Structural, optical and electrical properties of Hf-doped ZnO transparent conducting films prepared by sol–gel method. *J. Alloys Compd.* **2015**, *623*, 290–297, doi:10.1016/J.JALLCOM.2014.10.117.
6. Wang, G.H.; Shi, C.Y.; Zhao, L.; Diao, H.W.; Wang, W.J. Transparent conductive Hf-doped In 2 O 3 thin films by RF sputtering technique at low temperature annealing. *Appl. Surf. Sci.* **2017**, *399*, 716–720, doi:10.1016/j.apsusc.2016.11.239.
7. Alfakes, B.; Villegas, J.; Apostoleris, H.; Devarapalli, R.; Tamalampudi, S.; Lu, J.-Y.; Viegas, J.; Almansouri, I.; Chiesa, M. Optoelectronic Tunability of Hf-Doped ZnO for Photovoltaic Applications. *J. Phys. Chem. C* **2019**, *123*, 15258–15266, doi:10.1021/ACS.JPCC.9B02253.
8. Wang, G.H.; Shi, C.Y.; Zhao, L.; Mo, L.B.; Diao, H.W.; Wang, W.J. Efficiency improvement of the heterojunction solar cell using an antireflection Hf-doped In2O3 thin film prepared via glancing angle magnetron sputtering technology. *Opt. Mater. (Amst).* **2020**, *109*, 110323, doi:10.1016/J.OPTMAT.2020.110323.
